# ‘Man-in-the-barrel’ syndrome: a case report of bilateral arm paresis following cardiac arrest

**DOI:** 10.1097/MS9.0000000000000135

**Published:** 2023-02-08

**Authors:** Romana Riyaz, Syed A. Usaid, Niharikha Arram, Sumalatha Khatroth, Pranita Shah, Abhigan B. Shrestha

**Affiliations:** a Shadan Institute of Medical Sciences and Research; b Apollo Institute of Medical Sciences and Research; c Mallareddy Medical College for Women, Suraram, Hyderabad; d Prathima Institute of Medical Sciences and Research, Karimnagar, Telangana, India; e M Abdur Rahim Medical College, Dinajpur, Bangladesh

**Keywords:** brachial diplegia, cardiac arrest, man-in-a-barrel syndrome

## Abstract

**Background:**

‘Man-in-the-barrel syndrome’ (MIBS) is a neurological phenotype with brachial diplegia, normal sensation, and preserved motor function of the lower limb. Severe hypotension leading to watershed infarctions leading to this phenotype has been reported. The pathogenesis of MIBS is believed to be cerebral hypoperfusion leading to border zone infarctions between the territories of the anterior and middle cerebral arteries.

**Case Report and Discussion:**

A 49-year-old chronic alcoholic hypertensive Indian male was evaluated for barrel syndrome after a cardiac arrest. MRI confirmed hyperintensities between the territories of the anterior and middle cerebral arteries bilaterally.

**Conclusion:**

Person in barrel syndrome is a rare neurological syndrome. MIB is common after cerebral hypoperfusion and carries a poor prognosis. Identification of the underlying cause is important because the management and prognosis vary based on the etiology.

Highlights

- ‘Man-in-the-barrel syndrome’ is a rare neurological phenotype with brachial diplegia, normal sensation, and preserved motor function of the lower limb.
- The pathogenesis is believed to be cerebral hypoperfusion leading to border zone infarctions.
- Cardiac arrest can also lead to such a case; hence, prompt anticipation and management are required, as in our case described.

## Introduction

Barrel syndrome is a rare neurological phenotype with brachial diplegia with preserved motor function of the lower limb^1^. It was formerly called ‘Man in a barrel syndrome’ (MIBS) as it resembles the aspect of a patient being constrained in a barrel^1^. Various names were coined to describe the same clinical phenomenon over the years, such as distal field infarction^2^, cruciate paralysis^3^, and brachial amyotrophic diplegia syndrome^4^. Hypertensive emergencies are one of the main causes of end-organ damage leading to systemic hypoperfusion and ischemia at the border zones of anterior cerebral artery (ACA) and middle cerebral artery (MCA), subsequently causing barrel syndrome^5^,^6^.

We describe a case of ‘MIBS’ phenotype in a hypertensive patient following a precipitous drop in blood pressure due to cardiac arrest. This article has been delineated in accordance with the CAse REports (CARE) guideline^7^.

## Case presentation

We report a case of a 49-year-old chronic alcoholic hypertensive Indian male presenting with sudden onset of breathlessness at rest for 5 h and associated with palpitations. The patient presented via ambulance in the emergency department. The patient was found to be in acute left ventricular failure. There was no history of trauma, fever, cough, and peripheral edema.

On admission, a high blood pressure of 220/140 mmHg (normal: 140/90 mmHg) was observed. Shortly, he had sudden cardiac arrest, from which he was resuscitated after 15 min. Epinephrine was administered continuously as an intravenous infusion 1 mg every 3–5 min and antiedema measures like furosemide 1.0 mg/kg intravenously was injected slowly to prevent the progression of pulmonary edema. He required ventilatory support and remained unconscious and in shock for about 2 h. The blood pressure improved to 100/70 mmHg. The next day, postcardiac arrest, after regaining his consciousness completely, the patient complained of proximal upper limb weakness in the form of difficulty in abducting his arms.

On neurological examination, he had normal consciousness and no abnormal speech; his cranial nerve examination was normal, with normal bilateral fundi and no facial weakness. Upper limb examination presented as reduced tone, and according to the Medical Research Council (MRC), the power of proximal muscles was 3/5 with increased deep tendon reflexes and spasticity. Distal hand motor functions were normal with the normal sensory examination. Lower limb examination was unremarkable, with normal bilateral flexor plantar response and normal sensory examination.

**Figure 1:**
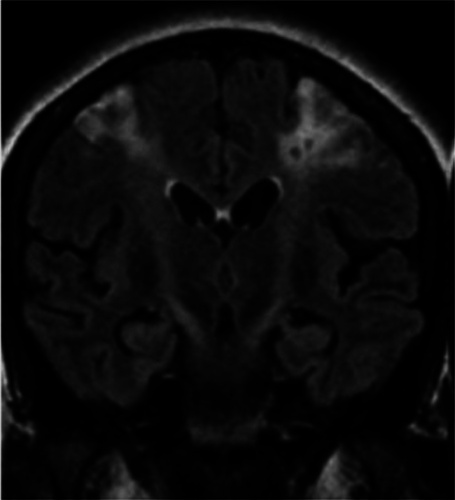
Plain MRI of the brain showing hyperintensity in bilateral watershed infarcts between bilateral anterior cerebral artery and middle cerebral artery territories.

**Figure 2:**
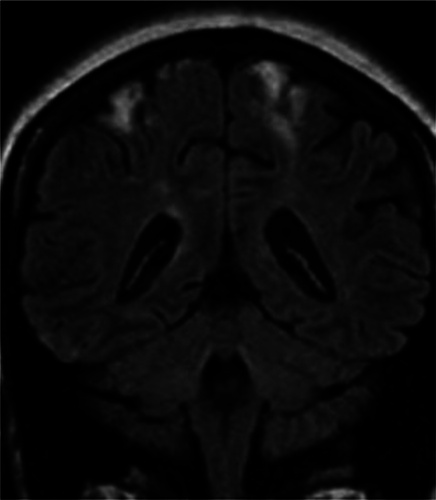
Plain MRI of the brain showing bilateral watershed infarcts between bilateral anterior cerebral artery and middle cerebral artery territories.

Chest X-ray was suggestive of cardiogenic pulmonary edema. ECG showed Q waves with V3, V4, V5, and V6, suggestive of myocardial infarction. Two-dimensional echocardiography showed regional wall motion abnormality in the anterior wall with dilated left atrium and ejection fraction of 33%, without the evidence of intracardiac thrombi or valvular dysfunction. A plain MRI of the brain revealed hyperintensity areas between a bilateral ACA and MCA suggestive of watershed infarcts (Figs 1, 2). Carotid artery duplex sonography was performed in bilateral carotid arteries and excluded carotid artery stenosis.

Blood investigations were performed to rule out other comorbid conditions, as shown in Table 1, and the anticoagulation profile was normal. Liver function tests were suggestive of ischemic hepatitis.

**Table 1:** Laboratory findings

Parameters	Results	Units	Reference values
Random blood sugar	130	g/dl	80–140
High-sensitivity troponin I (Abbott Assay)	1.8	ng/l	4–10 ng/l
Sodium	138	mmol/l	133–146
Potassium	3.9	mol/l	3.5–5.3
Creatinine	1.9	mg/dl	0.6–1.4
Urea	3.1	mmol/l	2.5–7.8
Total bilirubin	2.45	mg/dl	0.0–1.0
AST	187.9	U/l	0.0–50.0
ALT	92.4	U/l	0.0–45.0
Alkaline phosphatase	461.9	U/l	90.0–300.0
Total protein	5.15	g/dl	6.6–8.3
Albumin	3.7	g/dl	3.5–5.5

ALT, alanine transaminase; AST, aspartate transaminase.

Further, cardioprotective management included antiplatelet agents like aspirin 75 mg, statins like atorvastatin 40 mg, and physiotherapy was advised. The blood pressure was monitored at regular intervals. The liver function returns to normal within 3–4 days with correction of the underlying cause. He was extubated on day 4 and recovered from cardiogenic shock with residual bilateral weakness in his arms. After 3 days, the patient was fully recovered and released. However, follow-up could not be maintained due to a lack of patient compliance, probably due to the economic burden he was facing.

## Discussion

Under ACC/AHA guidelines, hypertension is diagnosed when a person’s systolic blood pressure in the office or clinic is at least 140 mmHg and/or diastolic blood pressure is at least 90 mmHg^8^. An estimated 7.5 million deaths per year, or roughly 12.8% of all deaths, are thought to be related to high blood pressure globally^9^.

Hypertension is associated with an increase in cardiac mortality and morbidity mainly due to a higher incidence of coronary artery disease and may also lead to left ventricular failure. During cardiac arrest, there is a sudden loss of cardiac pump function leading to a sudden drop in blood pressure and ultimately reduced cerebral perfusion. Preventing harmful arrest-related consequences on the brain is a unique challenge in cardiac arrest. More than half of individuals may experience some form of lasting brain damage if their entire circulatory arrest lasts longer than 5–8 min. Circulatory arrest lasting up to 10–15 min nearly usually results in considerable permanent loss of mental capacity.

Previously only one similar case has been reported by Shaw *et al*.^10^, in which they found MIBS as a sequel to cardiorespiratory arrest.

The brachial diplegia syndrome, known as ‘Man-in-the-barrel syndrome’ is uncommon but well-documented. The exact incidence of MIBS is unknown. In 1917, Dide and Lhermite^11^ gave the first description of it. The classic clinical presentation is paralysis of the upper extremity, more pronounced proximally than distally, with intact motor functions of the lower limbs. As the subject is unable to move his arms, it appears that the upper limbs are confined in a barrel^1^. In our case, the patient had proximal upper limb weakness but normal motor functions of distal hand muscles and lower limb muscles.

**Figure 3:**
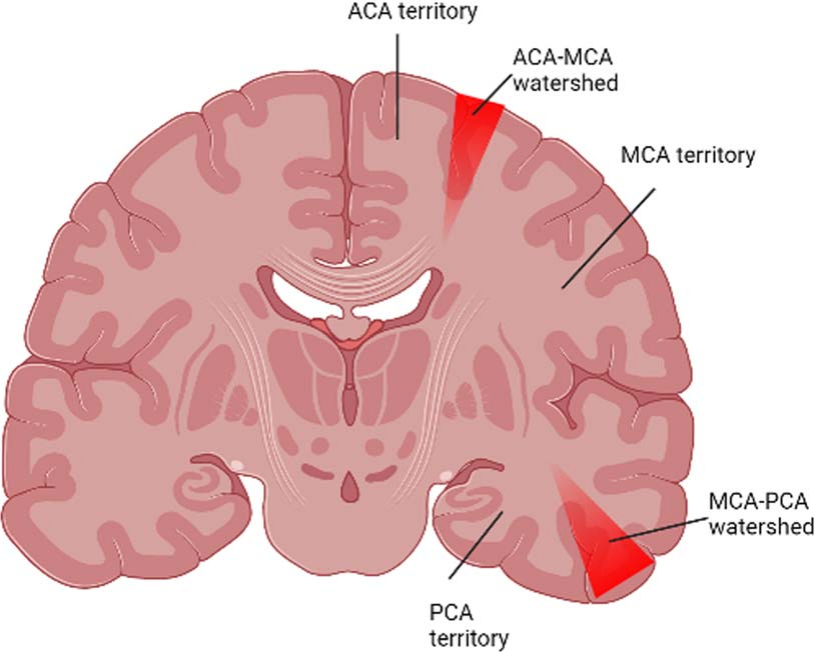
Anatomical area of the brain with watershed zones. ACA, anterior cerebral artery; MCA, middle cerebral artery; PCA, posterior cerebral artery.

Acute severe hypotension leads to hypoperfusion of the border regions of the cerebral vascular territories, which results in cell death/infarction, which is the cause of watershed strokes. Watershed strokes make up nearly 10% of all ischemic strokes 12. It usually happens between ACA and MCA. It is the place where the precentral gyrus, the motor area in charge of movement in the arm and shoulder, is located^13^. In our patient, an MRI of the brain revealed bilateral hyperintensities involving the watershed zones between ACA and MCA. Figure 3 shows the anatomical area of watershed zones of the brain.

Other alternative etiologies might result in the phenotypic manifestation of MIBS, as well. The motor cortex-watershed infarcts due to acute systemic hypotension (most frequent cause), multiple sclerosis, metastasis, brainstem-pontine myelinolysis, and spinal cord-anterior spinal artery infarct. Anterior horn cells-motor neuron disease, cervical nerve roots-bilateral C5, C6 radiculopathies, bilateral brachial plexus injury, peripheral nerves (multifocal motor neuropathy), neuromuscular junction (myasthenia gravis), or muscles (e.g. limb-girdle muscular dystrophy) can result in MIBS^1^,^14^.

The condition ‘Man-in-the-barrel syndrome’ is uncommonly reported and frequently goes undiagnosed by doctors. However, it might be more widespread than is generally believed. In our patient, the diagnosis is clear with a history of cardiac arrest for a prolonged time which suggests bilateral watershed infarcts as the cause of MIBS. The prognosis of this phenotype is quite variable, depending on the etiology. Some case series reported mortality up to 90% with motor neuron disease, whereas other cases point to a good recovery with minimal residual neurological deficit. Our patient had a satisfactory recovery due to timely diagnosis and treatment, along with improved weakness following physiotherapy.

## Conclusion

MIB is common after cerebral hypoperfusion, like cardiac arrest, and carries a poor prognosis. Timely identification of the underlying cause and localization of lesion(s) is important because the management and prognosis vary based on the etiology and site of the lesion.

## Ethical approval

Not applicable.

## Patient consent

Written informed consent was obtained from the patient for the publication of this case report and accompanying images. A copy of the written consent is available for review by the Editor-in-Chief of this journal on request.

## Sources of funding

Not sponsored.

## Author contribution

R.R.: conceptualization. All authors contributed equally.

## Conflicts of interest disclosure

There are no conflicts of interest.

## Research registration unique identifying number (UIN)

1. Name of the registry: not applicable.
2. Unique identifying number or registration ID: not applicable.
3. Hyperlink to your specific registration (must be publicly accessible and will be checked): not applicable.

## Guarantor

Abhigan Babu Shrestha.

## Provenance and peer review

Not commissioned, externally peer-reviewed.
